# *Drosophila melanogaster* tPlus3a and tPlus3b ensure full male fertility by regulating transcription of Y-chromosomal, seminal fluid, and heat shock genes

**DOI:** 10.1371/journal.pone.0213177

**Published:** 2019-03-07

**Authors:** Tim Hundertmark, Sabrina Kreutz, Nastasja Merle, Andrea Nist, Boris Lamp, Thorsten Stiewe, Alexander Brehm, Renate Renkawitz-Pohl, Christina Rathke

**Affiliations:** 1 Department of Biology, Developmental Biology, Philipps-Universität Marburg, Marburg, Germany; 2 Genomics Core Facility, Center for Tumor- and Immunobiology, Philipps-Universität Marburg, Marburg, Germany; 3 Institute of Molecular Oncology, Center for Tumor- and Immunobiology, Philipps-Universität Marburg, Marburg, Germany; 4 Institute of Molecular Biology and Tumor Research (IMT), Biomedical Research Centre (BMFZ), Philipps-Universität Marburg, Marburg, Germany; National Cancer Institute, UNITED STATES

## Abstract

Spermatogenesis in *Drosophila melanogaster* is characterized by a specific transcriptional program during the spermatocyte stage. Transcription of thousands of genes is regulated by the interaction of several proteins or complexes, including a tTAF-containing TFIID variant, tMAC, Mediator, and chromatin interactors, e.g., bromodomain proteins. We addressed how distinct subsets of target genes are selected. We characterized the highly similar proteins tPlus3a and tPlus3b, which contain a Plus3 domain and are enriched in the testis, mainly in spermatocytes. In *tPlus3a* and *tplus3b* deletion mutants generated using the CRISPR/Cas9 system, fertility was severely reduced and sperm showed defects during individualization. tPlus3a and tPlus3b heterodimerized with the bromodomain protein tBRD-1. To elucidate the role of the tPlus3a and tPlus3b proteins in transcriptional regulation, we determined the transcriptomes of *tplus3a-tplus3b* and *tbrd-1* deletion mutants using next-generation sequencing (RNA-seq) and compared them to that of the wild-type. tPlus3a and tPlus3b positively or negatively regulated the expression of nearly 400 genes; tBRD-1 regulated 1,500 genes. Nearly 200 genes were regulated by both tPlus3a and tPlus3b and tBRD-1. tPlus3a and tPlus3b activated the Y-chromosomal genes *kl-3* and *kl-5*, which indicates that tPlus3a and tPlus3b proteins are required for the function of distinct classes of genes. tPlus3a and tPlus3b and tBRD-1 repress genes relevant for seminal fluid and heat shock. We hypothesize that tPlus3a and tPlus3b proteins are required to specify the general transcriptional program in spermatocytes.

## Introduction

In mammals and in *Drosophila melanogaster*, regulation of transcription during spermatogenesis is complex. The germ cell transcriptional program covers various processes, including meiosis, post-meiotic formation of flagella, nuclear shaping, and chromatin reorganization. In flies, the majority of transcripts needed in spermatogenesis are produced in the prolonged meiotic prophase of the spermatocyte stage. Therefore, most transcripts required for post-meiotic sperm development (spermiogenesis) are translationally repressed until they are needed in later stages [1–4]. About 50% of the predicted genes in the *D*. *melanogaster* genome are expressed in the testis [5], and a large portion of the transcripts are testis enriched or even testis-specific.

One complex that plays a pivotal role in transcriptional regulation during spermatogenesis is a germ-line-specific variant of the general transcription factor TFIID. This complex is composed of somatic TATA-binding protein (TBP)-associated factors (TAFs) and the testis-specific paralogues tTAFs [6–7]. tTAFs are recruited by the Mediator complex to chromatin, where they activate genes [8]. Mediator regulates several hundred genes, and some of its subunits are recruited to gene regions by the testis meiotic arrest complex (tMAC), which is involved in regulation of spermiogenesis-relevant genes [8–9]. Together, tTAFs, tMAC, and Mediator regulate thousands of genes [3].

It is assumed that besides these general transcription initiation factors, other factors target gene expression more specifically. These could include proteins that interact with the general transcription complex, particularly because upstream regulatory regions of genes specifically expressed in the male germ line are often very short [10]. Indeed, bromodomain-containing proteins (tBRDs), which are synthesized specifically in the testis, heterodimerize with some of the tTAFs and partly with each other [11–12]. In general, bromodomain proteins bind to acetylated lysine residues [13], which suggests that tBRDs might serve as a platform to guide parts of the transcriptional machinery to chromatin. The well-characterized tBRD-1 and tBRD-2 proteins regulate smaller sets of target genes than tTAFs and tMAC, and both depend on tTAF function. Loss of *tbrd-1* or knock-down of *tbrd-2* leads to post-meiotic phenotypes [11–12, 14], whereas *tTAF* mutants show an earlier meiotic arrest phenotype [6]. Despite heterodimerization of tBRD-1 and tBRD-2 and a similar phenotype of *tbrd-1-* and *tbrd-2*-deficient flies, microarray analyses suggest that both proteins are also part of different subcomplexes, as they regulate partially non-overlapping sets of target genes [12].

We aimed at identifying further transcriptional regulators and at clarifying whether they physically interact with one of the known tBRD molecules and share target genes. As candidates, we considered Rtf-related proteins synthesized specifically in the testis. RTF is a member of a complex that is conserved in yeast, mouse, human, and fly. Subunits of this complex—the RNA-polymerase-II-associated factor complex (Paf1C) [15]—co-purify with RNA polymerase II subunits in yeast [16] and higher eukaryotes [15, 17]. Many studies have suggested that Paf1C plays a role in transcriptional regulation, and several mechanisms have been proposed on how the effect on transcription might be achieved, including transcription elongation, RNA polymerase II pausing, or maintenance of co-transcriptional histone modifications [18]. Some subunits of Paf1C are conserved, namely Paf1, Cdc73, Leo1, Ctr9, and Rtf1 [15]. Rtf1 can also function outside of the Paf1C complex in humans and *D*. *melanogaster* [17, 19–21]. Rtf1 contains several functional domains, including a histone modification domain, a Plus3 domain, and a protein interaction domain that mediates Paf1C interaction. It has been suggested that in yeast, the histone modification domain mediates co-transcriptional histone modifications and the Plus3 domain is needed for Paf1C recruitment to chromatin [15, 22]. Strikingly, the Plus3 domain is highly conserved in yeast, human, and fly. Structural homology and *in vitro* experiments using recombinant Rtf1 indicate that the Plus3 domain binds nucleic acids, especially single-stranded DNA. This feature suggests a supportive role in transcription through stabilization of the transcription machinery during transcript elongation [23]. However, evidence for an interplay between the complexes involved in transcriptional regulation is fragmentary. In *D*. *melanogaster*, Rtf1 is ubiquitously transcribed [5]. In a search for proteins that might play a role in refining the transcriptional program of spermatocytes in *D*. *melanogaster*, we investigated proteins carrying the Plus3 domain that are mainly synthesized in the testes. Indeed, in a stage-specific proteome analysis, we showed that tPlus3a and tPlus3b proteins are highly enriched in stages before meiotic divisions [24]. Here, we characterized tPlus3a and tPlus3b and show that they are essential for spermiogenesis and full male fertility.

## Results

### Identification of three genes specifically expressed in the male germ line that encode Plus3 domain proteins

**Fig 1:**
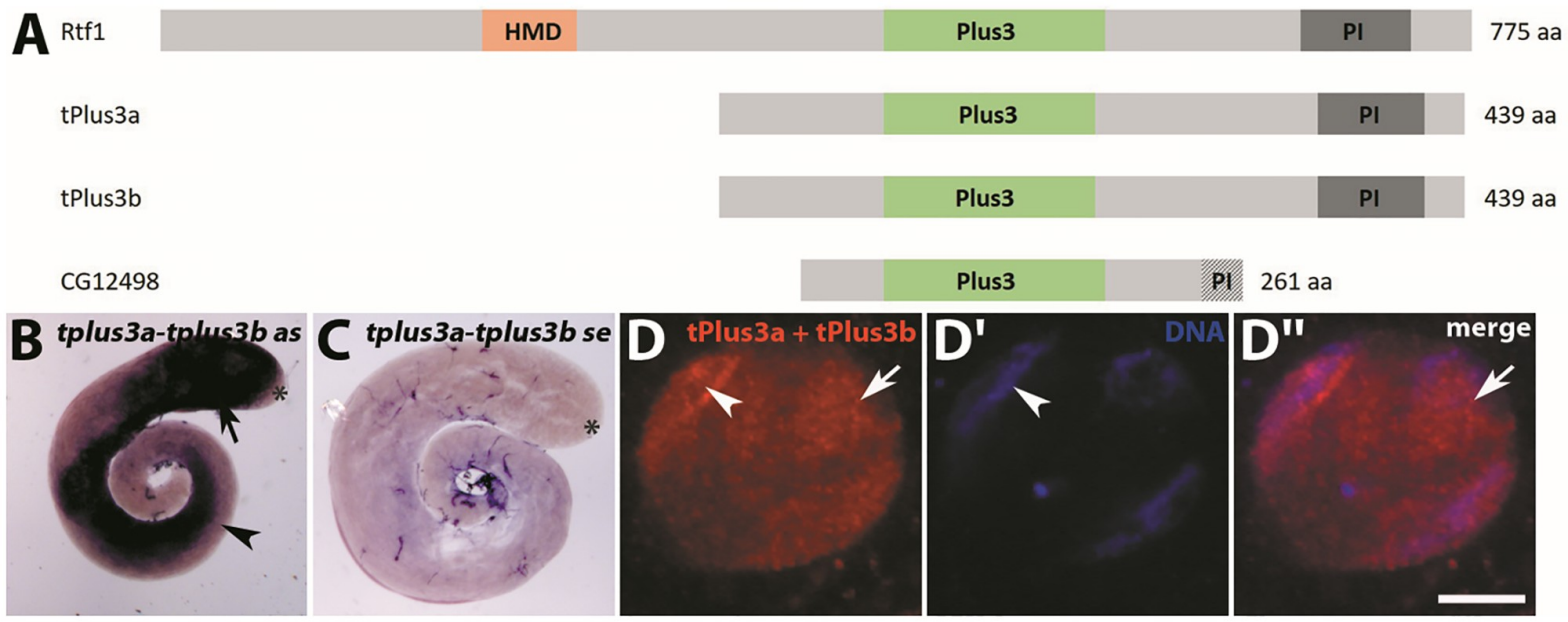
tPlus3a and tPlus3b proteins localize mainly to the nuclei of spermatocytes. Schematic view of the alignment of the *D*. *melanogaster* Plus3 domain-containing proteins Rtf1, tPlus3a, tPlus3b, and CG12498. aa, length of protein in amino acids; HMD, histone modification domain; Plus3, Plus3 domain; PI, protein interaction domain. (B, C) *In situ* hybridization of wild-type testis with (B) *tplus3a-tplus3b* antisense (as) probe and (C) *tplus3a-plus3b* sense (se) probe. Asterisk, hub region; arrow, detection of *tplus3a-tplus3b* mRNA in spermatocytes; arrowhead, lack of detection in late stages. (D) Single spermatocyte nucleus from wild-type testes stained with anti-tPlus3a and tPlus3b antibody. Arrowhead, tPlus3a and tPlus3b distributed throughout nucleus, but slightly concentrated in chromosomal regions; arrow, tPlus3a and tPlus3b in distinct areas between chromosomal regions. (D’) DNA visualized with Hoechst 33258. (D”) Merged image of D and D’. Scale bar = 5 μm.

During spermatogenesis in *D*. *melanogaster*, transcription mainly takes place in spermatocytes, where distinct complexes regulate thousands of genes. We aimed at identifying testes-specific regulators responsible for distinct subsets of genes. One of the known general complexes that are involved in transcriptional regulation is Paf1C [15]. A prominent subunit of Paf1C is the Plus3 domain protein Rtf1; Rtf1 and especially its Plus3 domain are conserved in *Saccharomyces cerevisiae*, *D*. *melanogaster*, and *Homo sapiens* [23]. In *D*. *melanogaster*, the gene is ubiquitously expressed and is highly expressed in the testis. In a search for genes of other proteins with a Plus3 domain that are specifically expressed in the *D*. *melanogaster* testis, we identified three genes, namely *CG12498*, *CG31702*, and *CG31703*. According to RNA-seq and microarray analyses, all three genes are mostly expressed in the testis [5, 25]. The nucleotide sequences of *CG31702* and *CG31703* are approximately 99% identical. Based on their testis-specific transcription and their encoded Plus3 domain, we named the *CG31702* and *CG31703* genes *tplus3a* and *tplus3b* (testis-specifically transcribed *plus3* genes), respectively. The proteins encoded by *CG12498*, *tplus3a*, and *tplus3b* share conserved regions (Fig 1 and S1 Fig). Due to the high similarity between tPlus3a and tPlus3b we call them tPlus3a and tPlus3b in the following. The Plus3 domains of tPlus3a and tPlus3b are identical. tPlus3a and CG12498 contain the three conserved positively charged amino acids that gave the domain its name (green in S1 Fig) and many other amino acids that are crucial for domain conformation [23]. We used Clustal Omega [26] to align the amino acid sequences of Rtf1, tPlus3a, and CG12498 (S1A Fig). The histone modification domain (HMD in Fig 1A) of Rtf1 is not present in the other Plus3 domain proteins. All four proteins have a conserved sequence near the C-terminus; in Rtf1, this sequence serves as a protein interaction domain (PI in Fig 1A). This domain is truncated in CG12498, thus it is functionality may be severely impaired (S1B Fig). The Plus3 domain sequence in tPlus3a is slightly shorter than that in Rtf1 and CG12498 (S1A Fig).

### *tplus3a-tplus3b* transcripts are expressed and their proteins are synthesized in male germ cells

We used RNA *in situ* hybridization to examine the distribution of *tplus3a-tplus3b*, *CG12498*, and *Rtf1* transcripts in the testis of wild-type flies (Fig 1B and S2A and S2C Fig). *tplus3a-tplus3b* transcripts were detected from the spermatocyte stage onward (Fig 1B), but late stages of spermatogenesis did not show any signal (Fig 1B). Thus, *tplus3a-tplus3b* transcripts characterize the spermatocyte and early spermatid stage. A control probe containing the sense sequence did not yield any signal (Fig 1C). *CG12498* transcripts were present mainly in stages before meiotic divisions, while *rtf1* transcripts were visualized in addition in post-meiotic stages (S2A and S2C Fig). We unsuccessfully attempted to generate flies that synthesized CG12498-mCherry to analyze the subcellular localization of CG12498. However, we were able to generate transgenic *tplus3b-eGFP* flies; tPlus3b-eGFP was expressed in the nucleoplasm of spermatocytes (S3A Fig, arrow), and the nucleolus was labeled (S3A Fig, arrowhead). In agreement with the transcript signal, we observed tPlus3b-eGFP in nuclei of round spermatids (S3B Fig, double arrow).

In addition, we raised a peptide antibody that could detect tPlus3a and tPlus3b. In immunofluorescence stainings of squash preparations of wild-type testes, tPlus3a and tPlus3b proteins were detected only in the nuclei of spermatocytes. In contrast to visualization with tPlus3b-eGFP, the nucleolus was not labeled. This lack of labeling might be due to inaccessibility of the antigenic region of tPlus3a and tPlus3b for the antibody or to artificial localization of eGFP in the nucleolus. The signal was fairly homogenously distributed in the nucleus and slightly concentrated in chromosomal regions (Fig 1D–1D”) and in distinct areas between the chromosomal regions in the nucleus (Fig 1D and 1D”). This region between the Hoechst-positive chromosomal regions contains large lampbrush loops, which correspond to three of the Y-chromosomal fertility loci [27]. We conclude that tPlus3a and tPlus3b expression is characteristic for the highly transcriptionally active spermatocyte phase.

### tPlus3a and tPlus3b proteins are required for full male fertility

**Fig 2:**
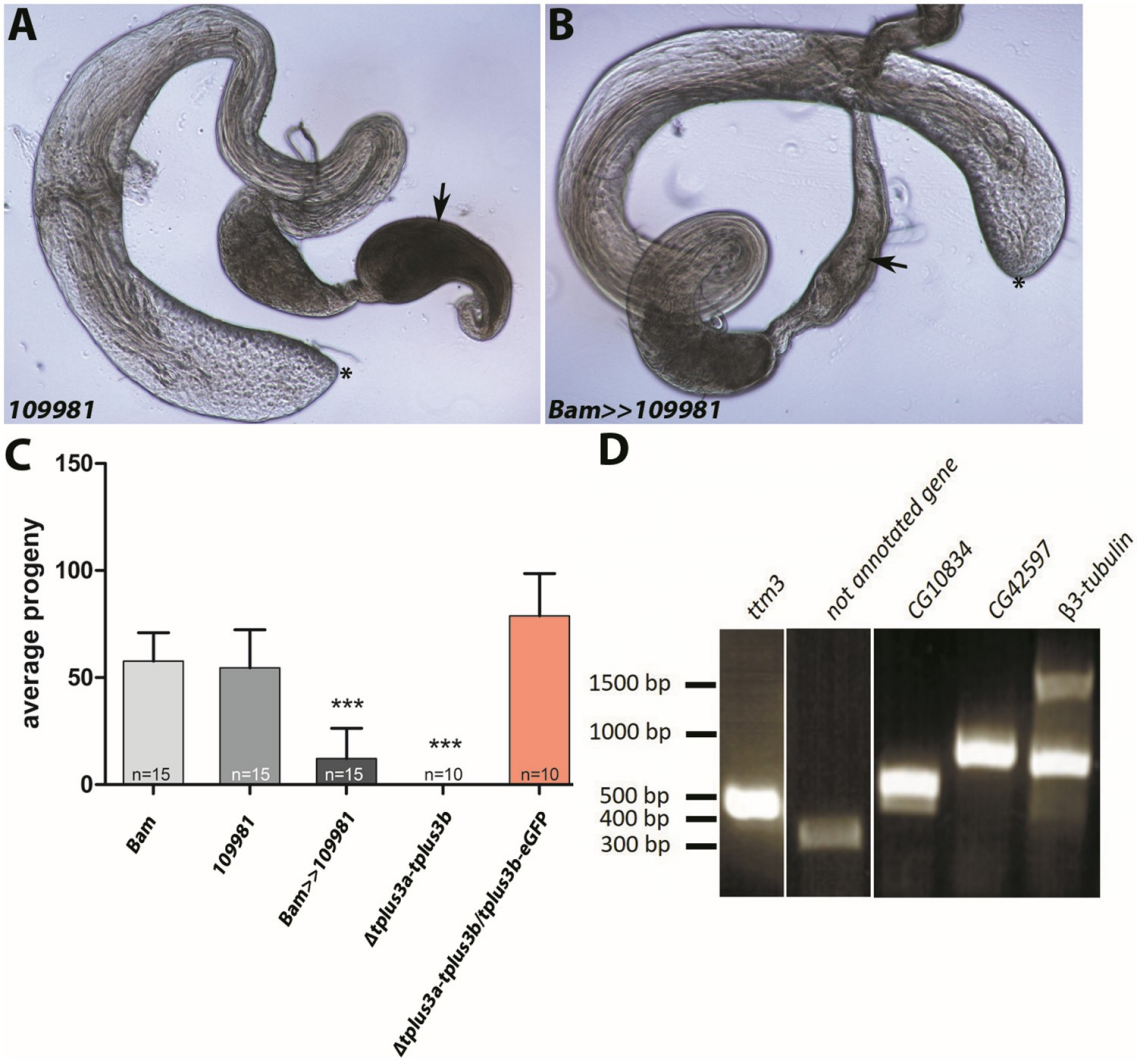
Knock-down of *tplus3a-tplus3b* leads to severely reduced male fertility. (A) Seminal vesicle (arrow) of a male of the undriven *tplus3a-tplus3b* RNAi line filled with sperm. (B) Seminal vesicle (arrow) of a male from the bam GAL4 driven RNAi line v109981 contains few sperm. (C) Fertility tests of *tplus3a-tplus3b* knock-down males and sterility rescue of homozygous *Δtplus3a-tplus3b* mutants by tPlus3b-eGFP. (D) Analysis of genomic DNA of *Δtplus3a-tplus3b* mutants showed the presence of *ttm3*, a not-annotated gene, *CG10834*, *CG42597*, and the *β3-tubulin* gene as control. Dashes indicate the position of marker ladder.

To determine whether tPlus3a and tPlus3b proteins are essential for male fertility, we first used an RNAi approach to reduce the transcript levels of *tplus3a-tplus3b* (Fig 2). The analysis of testes and seminal vesicles of male flies revealed that the seminal vesicles of knock-down males (Fig 2B, arrow) contained much less sperm than seminal vesicles of control males (Fig 2A, arrow). This is in agreement with strongly reduced fertility of *tplus3a-tplus3b* knock-down males (Fig 2C).

As these data suggested a crucial role of *tplus3a-tplus3b* in male fertility, we generated deletion mutants using the CRISPR/Cas9 system. We established transgenic flies carrying a construct coding for a single guide RNA able to detect both *tplus3a* and *tplus3b* and crossed these with flies expressing Cas9 in the germ line [28]. We screened 50 independent single crossings of putative mutants via PCR and identified the deletion mutant *Δtplus3a-tplus3b*, in which large parts of the open reading frame (ORF) of both *tplus3a* and *tplus3b* were deleted. We did not obtain any mutants with solely a *tplus3a* or *tplus3b* deletion. The deletions led to a fusion of *tplus3a* within its ORF to *CR31700*, an inactive pseudogene approximately 4,000 base pairs (bp) upstream of *tplus3a* [5, 25] and yielded a null allele of *tplus3a*. In addition, it was possible to PCR amplify regions flanking *tplus3b* but not its ORF, which indicated that most of the *tplus3b* ORF was deleted. Thus, this mutant likely carries loss-of-function mutations for both genes; the genes neighboring *tplus3a* and *tplus3b*, other than *CR31700*, remained intact (Fig 2D), this also held true for the non-annotated gene with expression in accessory glands (modENCODE). The *tplus3b-eGFP* construct was able to strongly reduce sterility of homozygous *Δtplus3a-tplus3b* mutants (Fig 2C). This rescue indicated, that mainly the loss of *tplus3a and tplus3b* was responsible for the observed sterility defects and that *tplus3b-eGFP* can rescue sterility, which might argue for functional redundancy between tPlus3a and tPlus3b.

**Fig 3:**
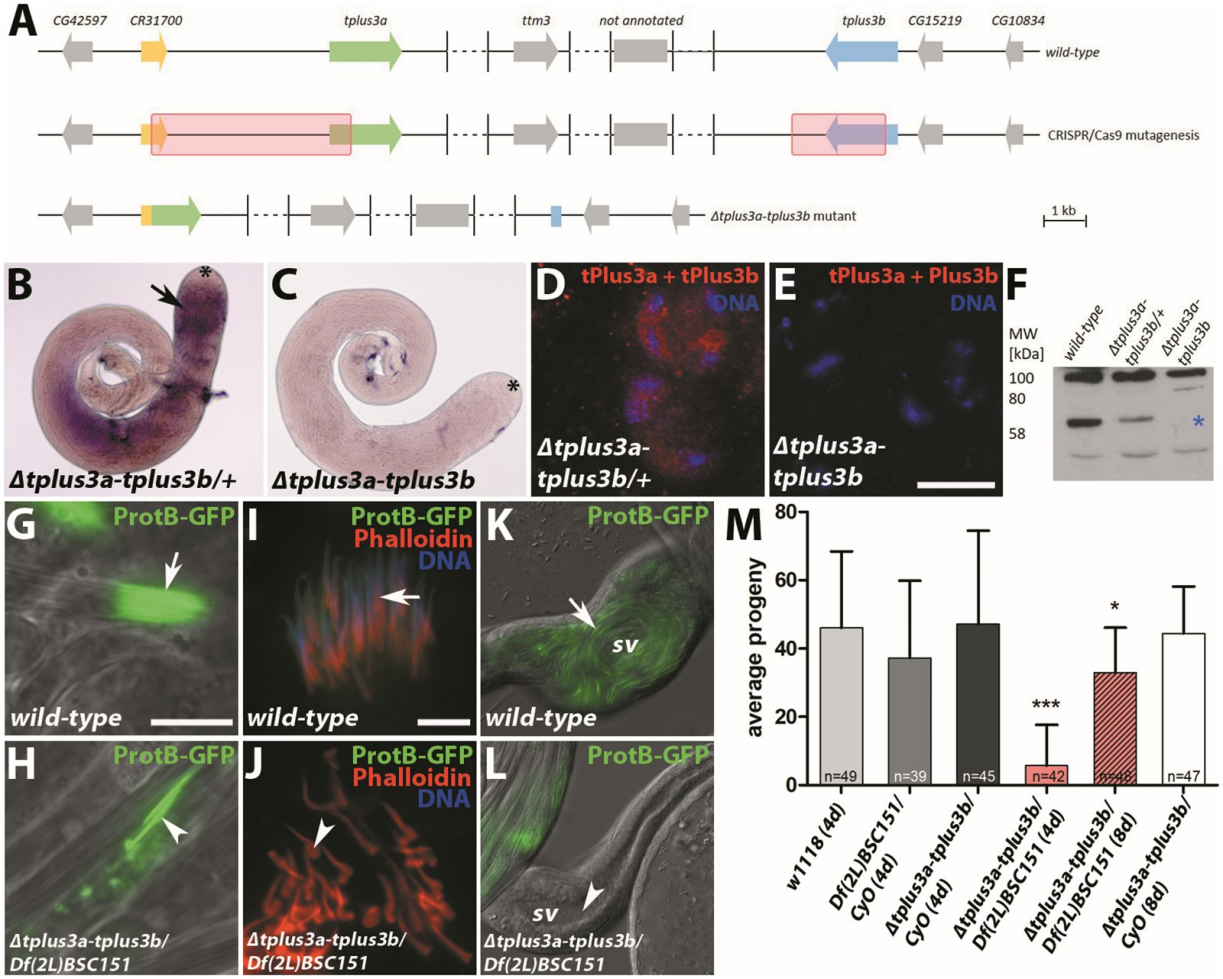
Deletion of *tplus3a-tplus3b* leads to severely reduced male fertility and defects during sperm individualization. (A) Schematic view of the *tplus3a and tplus3b* genomic region and the CRISPR/Cas9-mediated generation of deletions (red boxes), which resulted in the fusion of *tplus3a* and pseudogene *CR31700* and a deletion of most of the *tplus3b* ORF. Dashed lines, regions not fully depicted. The region between *ttm3* and *tplus3b* contains a not-annotated gene which is expressed in the accessory glands of the male reproductive track (modENCODE). (B, C) *In situ* hybridization of testis of (B) heterozygous *ΔpPlus3a-tplus3b* mutant (arrow, *tplus3a-tplus3b* signal detected) and (C) homozygous *Δtplus3a-tplus3b* mutant. Asterisk, hub region. (D, E) Spermatocytes of (D) heterozygous and (E) homozygous *Δtplus3a-tplus3b* mutant testes stained with anti-tPlus3a and tPlus3b. Scale bar = 10 μm. (F) Western blot of protein extracts from wild-type and homozygous and heterozygous *Δtplus3a-tplus3b* mutant testes probed with anti-tPlus3a and tPlus3b. Asterisk, lack of signal at ca. 60 kDa. (G, H) Whole-mount preparations of (G) wild-type and (H) *Δtplus3a-tplus3b/Df(2L)BSC151* testes. Sperm nuclei visualized with ProtB-eGFP. Arrow, sperm nuclei aligned in tight bundles; arrowhead, sperm nuclei distributed across the testis. Scale bar = 10 μm. (I, J) Squash preparations of (I) wild-type and (J) *Δtplus3a-tplus3b/Df(2L)BSC151* testes. Sperm nuclei visualized with ProtB-eGFP, DNA stained with Hoechst 33258, and individualization complex stained with TRITC-Phalloidin. Arrow, sperm nuclei aligned in tight bundles; arrowhead, disturbed individualization complex formation with one end thickened. Scale bar = 10 μm. (K, L) Whole-mount preparations of (K) wild-type and (L) *Δtplus3a-tplus3b/Df(2L)BSC151* seminal vesicles (sv) of 1-day-old males. Sperm nuclei visualized with ProtB-eGFP. Arrow, seminal vesicles filled with sperm; arrowhead, empty seminal vesicles. (M) Fertility of 4-day-old wild-type, 4-day-old *Df(2L)BSC151/CyO*, 4- and 8-day-old *Δtplus3a-tplus3b/CyO*, and 4- and 8-day-old *Δtplus3a-tplus3b/Df(2L)BSC151* flies. *, p < 0.05; ***, p < 0.005.

*In situ* hybridization detected transcripts in testis of heterozygous *Δtplus3a-tplus3b* mutants (Fig 3B, arrow) but not in homozygous mutants (Fig 3C). Analogous results were obtained in anti-tPlus3a and tPlus3b immunofluorescence stainings. tPlus3a and tPlus3b proteins were detected in spermatocytes of heterozygous *Δtplus3-tplus3b* mutant flies (Fig 3D) but not in homozygous *ΔtPlus3a-tplus3b* mutants (Fig 3E).

In western blots using protein extracts from testes and probed with anti-tPlus3a and tPlus3b, a prominent band at approximately 60 kDa was detected in the wild-type (predicted molecular mass of tPlus3a and tPlus3b ca. 50 kDa). The signal of protein extracts of heterozygous *Δtplus3a-tplus3b* testes was lower, and no signal was detected when protein extracts of homozygous *Δtplus3a-tplus3b* testes were probed (Fig 3F).

To exclude off-target effects arising during CRISPR/Cas9 mutagenesis, we crossed *Δtplus3-tplus3b/CyO* with *Df(2L)BSC151/CyO*, which is a fly line deficient in *tplus3a* and *tplus3b*. All further phenotypic analyses were carried out with *trans*-heterozygous *Δtplus3a-tplus3b/Df(2L)BSC151* flies. These flies were mildly defective in sperm individualization compared to wild-type flies; in wild-type flies, the nuclei of spermatids in an individual cyst with synchronously developing spermatids are arranged strictly parallel, as visualized with *protB-eGFP* transgene expression (Fig 3G). In *Δtplus3a-tplus3b/Df(2L)BSC151* flies, many sperm bundles were arranged in parallel, but some sperm were dispersed in the testis and lost their cyst organization (Fig 3H). In wild-type testis squash preparations, many cysts appeared and the individualization complex formed normally, as visualized with TRITC-phalloidin (Fig 3I). In *Δtplus3a-tplus3b/Df(2L)BSC151* testis squash preparations, a number of sperm bundles were disorganized. In some cases, the individualization complex formed was abnormal in that one end was thickened (Fig 3J). The seminal vesicles of wild-type flies were already filled with sperm 1 day after hatching (Fig 3K, arrow), whereas the seminal vesicles of *Δtplus3a-tplus3b/Df(2L)BSC151* males of the same age were empty (Fig 3L, arrowhead). Two days after hatching, also seminal vesicles of *Δtplus3a-tplus3b/Df(2L)BSC151* males contained sperm, the number of sperm in the seminal vesicles increased with age as exemplarily shown in S4C and S4D Fig in comparison to wild-type (S4A and S4B Fig). For fertility assays, we mated one day old males and analyzed the number of progeny after further 3 days (4 day old males), alternatively, we mated 5 day old males with virgins and analyzed the number of progeny after 3 days (8 day old males). In these sterility tests comparing 4-day-old wild-type, *Df(2L)BSC151/CyO*, *Δtplus3a-tplus3b/CyO*, and *Δtplus3a-tplus3b/Df(2L)BSC151* males, the fertility of *Δtplus3a-tplus3b/Df(2L)BSC151* males was reduced to ca. 12% of that of the wild-type control (Fig 3M). As 2-day-old *Δtplus3a-tplus3b/Df(2L)BSC151* males started to have sperm in the seminal vesicles, the reduced fertility after 4 days suggests that sperm found in the seminal vesicles were less than in wild-type males or have reduced in motility. Sterility tests with 8-day-old *Δtplus3a-tplus3b/Df(2L)BSC151* males revealed that fertility is partially restored after several days (Fig 3M). We conclude that defects in late sperm development result in delayed fertility of *Δtplus3a-tplus3b/Df(2L)BSC151* males and that the proportion of sperm in *Δtplus3a-tplus3b/Df(2L)BSC151* males increases with age. At first glance, this was surprising, but lack of *tplus3a-tplus3b w*as possibly compensated by the increase in synthesis of the *CG12498*-encoded tPlus3 domain protein or by dRtf1, which in contrast to CG12498 contains a complete PI domain (Fig 1A).

### tPlus3a and tPlus3b heterodimerize with the bromodomain protein tBRD-1

**Fig 4:**
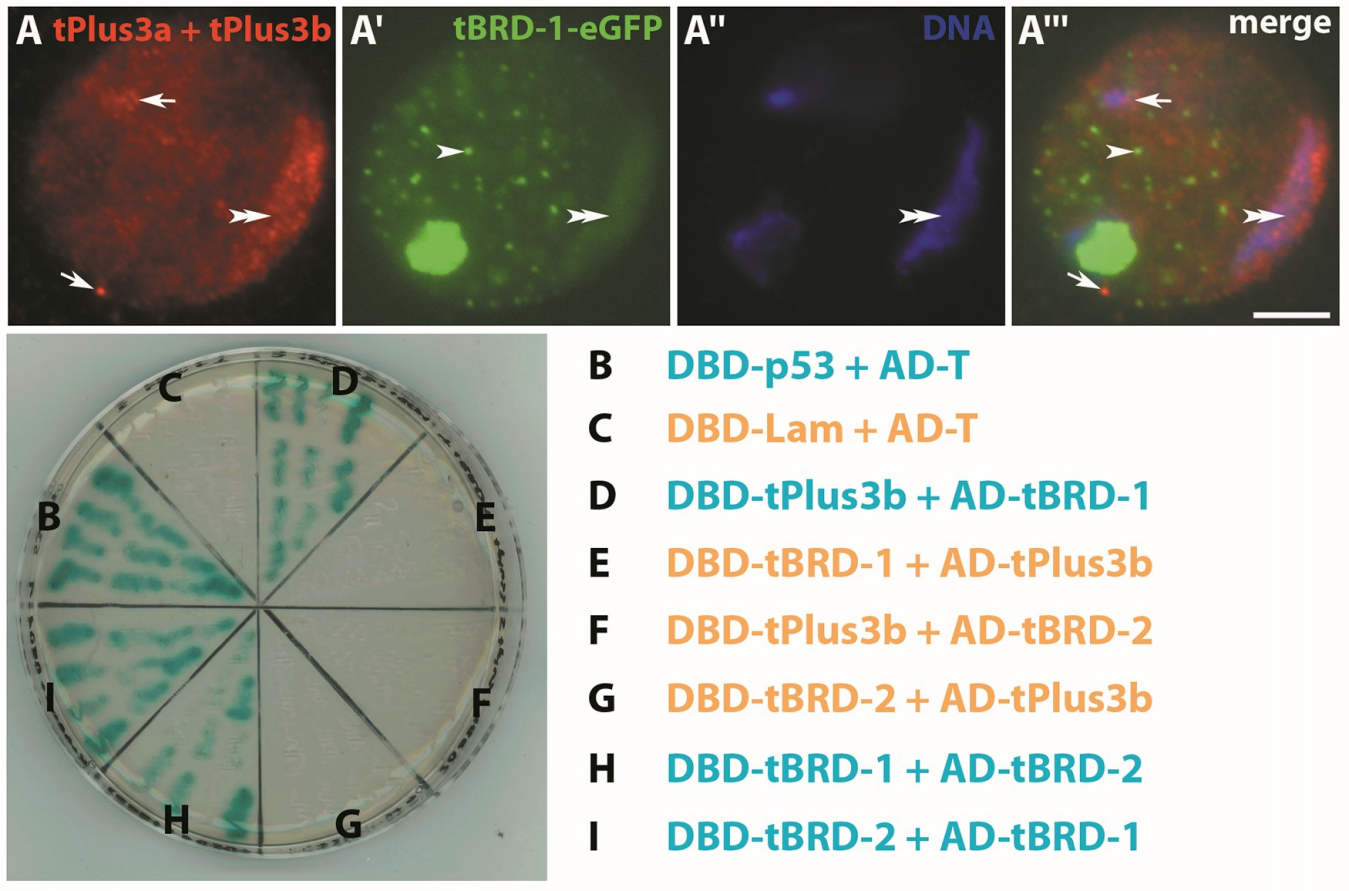
tPlus3a and tPlus3b partially co-localize with tBRD-1 and interact with tBRD-1 but not with tBRD-2 in yeast two-hybrid experiments. (A–A”‘) Anti-tPlus3a and tPlus3b immunofluorescence staining of a single spermatocyte nucleus of flies synthesizing tBRD-1-eGFP. (A) tPlus3a and tPlus3b signal present throughout the nucleus but concentrated in chromosomal regions (double arrowhead) and in distinct spots (arrows). (A’) tBRD-1 found mainly in the nucleolus, in chromosomal regions (double arrowhead), and in a speckled pattern (arrowhead). (A”) DNA stained with Hoechst. Double arrowhead, chromosomal regions. (A”‘) Merged image of A, A’, and A” showing co-localization of tPlus3a and tPlus3b and tBRD-1-eGFP in the chromosomal regions (double arrowhead). Scale bar = 5 μm. (B–I) Yeast two-hybrid interaction assay. (B) Positive control. (C) Negative control. (D) Heterodimerization of tPlus3b bait and tBRD-1 prey. (E) Lack of heterodimerization of tPlus3b prey and tBRD-1 bait. (F, G) Lack of heterodimerization of tPlus3b as bait or prey and tBRD-2 as prey or bait. (H, I) Heterodimerization of tBRD-1 and tBRD-2 as prey or bait.

We then addressed whether tPlus3a and tPlus3b proteins interact with other known proteins of transcription complexes in spermatocytes. We previously observed a late arrest in spermatogenesis and infertility of *tbrd-1* mutants and after *tbrd-2* knock-down in male germ cells [12, 14]. We analyzed tPlus3a and tPlus3b immunofluorescence stainings at high magnification in flies synthesizing tBRD-1-eGFP to determine whether tPlus3a and tPlus3b co-localize with tBRD-1 in nuclei of spermatocytes (Fig 4A–4A”‘). The tBRD-1-eGFP signal was concentrated in the chromosomal regions (Fig 4A’) and overlapped with the tPlus3a and tPlus3b staining in this region (Fig 4A and 4A”‘). The tBRD-1-eGFP signal also concentrated in speckles widely distributed in the nucleus (Fig 4A’), but these areas did not overlap significantly with the prominent tPlus3a and tPlus3b staining between the chromosomal regions (Fig 4A and 4A”‘).

The partial co-localization of tPlus3a and tPlus3b and tBRD-1 in the chromosomal regions prompted us to test whether tBRD-1 and also tBRD-2, whose synthesis overlaps with that of tBRD-1, interact with tPlus3a and tPlus3b. We analyzed whether tPlus3a and tPlus3b heterodimerize with tBRD-1 or tBRD-2 using yeast two-hybrid assays. The coding regions of tPlus3b, tBRD-1, and tBRD-2 were fused to the GAL4 DNA binding domain (DBD), which represented the bait protein, and to the GAL4 activation domain (AD), which represented the prey protein. Interaction of bait and prey fusion proteins leads to activation of a MEL1 reporter gene, which stains yeast colonies blue. The positive control, with DBD-p53 as bait protein and the large T antigen (AD-T) as prey protein, activated the reporter gene (Fig 4B), whereas the negative control, with Lamin C as bait protein and the large T antigen as prey protein, did not (Fig 4C).

In the assay, the bait fusion DBD-tPlus3b heterodimerized with the prey fusion AD-tBRD-1 (Fig 4D). To exclude auto-activation of reporter genes that might be caused by bait or prey fusion proteins, we tested DBD-tPlus3b with an unfused activation domain; the reporter gene was not activated (not shown). We did not detect heterodimerization of DBD-tBRD-1 with AD-tPlus3b (Fig 4E), possibly because of conformational changes in the tPlus3b structure caused by the fusion to the activation domain or insufficient synthesis of the AD-tPlus3b fusion. DBD-tPlus3b did not heterodimerize with AD-tBRD-2 (Fig 4F), and DBD-tBRD-2 did not heterodimerize with AD-tPlus3b (Fig 4G). As a control for the functionality of the yeast two-hybrid constructs, we tested the previously described heterodimerization of tBRD-1 and tBRD-2 [11]; the proteins formed a heterodimer (Fig 4H and 4I).

### tPlus3a and tPlus3b and tBRD-1 activate and repress transcription individually and together

**Fig 5:**
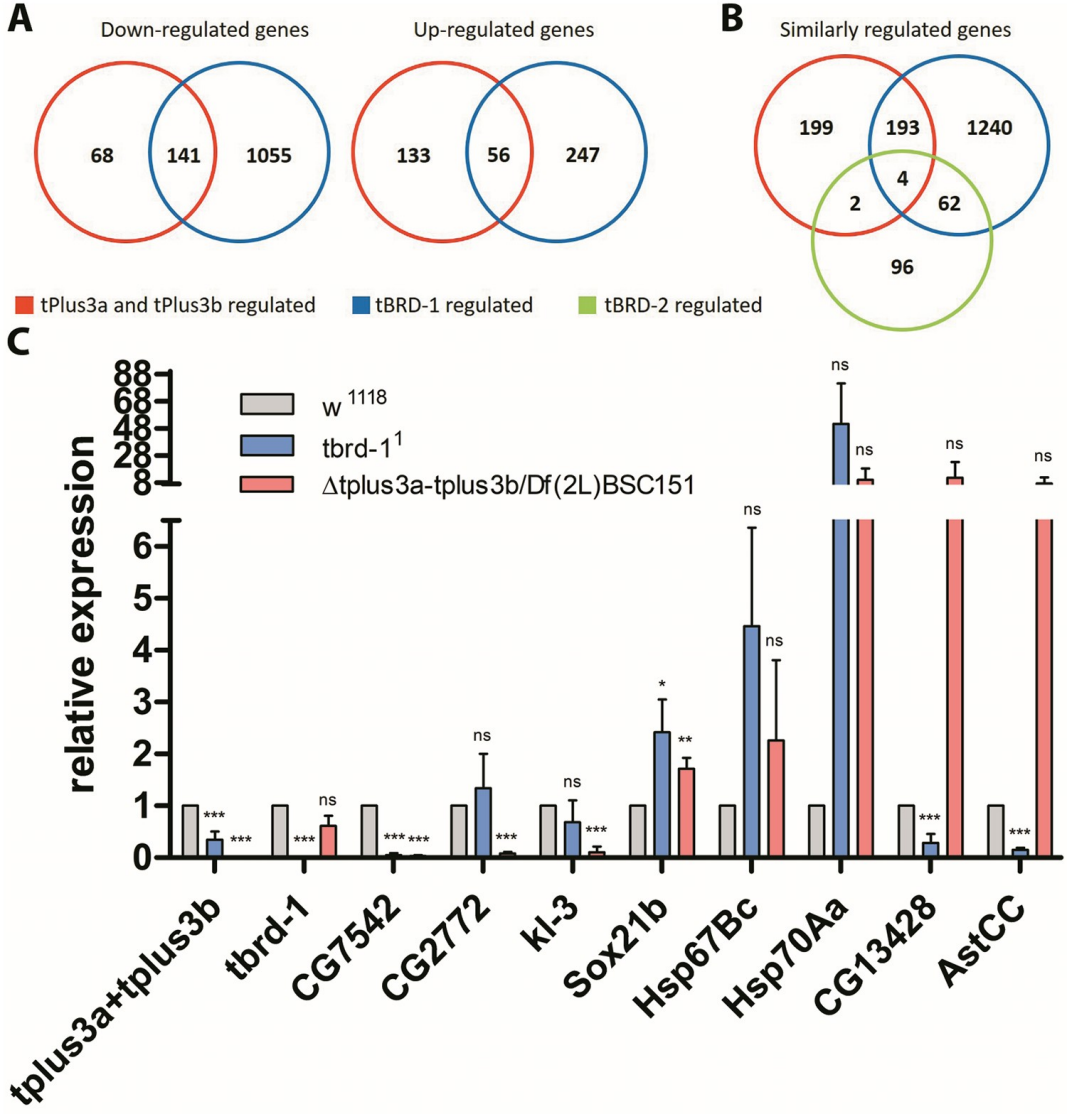
tPlus3a and tPlus3b and tBRD-1 regulate a common set of genes. Transcript expression was measured with RNA-seq in three replicates each of testes RNA of the wild-type, *Δtplus3a-tplus3b/Df(2L)BSC151*, and *tbrd-1*^*1*^ mutants. Expression levels in mutants are relative to those in the wild-type, which were set to 1. Differentially expressed genes were identified using a log2 FC ≥ 1 and ≤ −1. (A-C) Numbers indicate the number of genes that were shown to be regulated (A) Venn diagrams showing the overlap between down-regulated and up-regulated genes in *Δtplus3a-tplus3b/Df(2L)BSC151* (tPlus3a and tPlus3b regulated) and *tbrd-1*^*1*^ (tBRD-1 regulated) mutants relative to the wild-type. (B) Venn diagram showing the overlap of similarly regulated genes in *Δtplus3a-tplus3b/Df(2L)BSC151*, *tbrd-1*^*1*^, and *tbrd-2* knock-down testes (tBRD-2 regulated). (C) Validation of RNA-seq results using qPCR, normalized to Rpl32. As controls, no *tplus3a-tplus3b* and *tbrd-1* transcripts were expressed in the respective mutants. P-values for significance *Δtplus3a-tplus3b/Df(2L)BSC151* or *tbrd-1*^*1*^ compared to wild-type: * p < 0.025, ** p < 0.005 and *** p < 0.0025.

Next, we addressed whether tPlus3a and tPlus3b proteins are important for gene activation and/or repression. As our yeast two-hybrid results showed that tPlus3b and tBRD-1 form a heterodimer, we asked whether tPlus3a and tPlus3b and tBRD-1 share target genes. We sequenced testis RNA from *Δtplus3a-tplus3b/Df(2L)BSC151* flies, *tbrd-1*^*1*^ mutants, and wild-type flies in parallel and identified differentially expressed genes (log2 FC ≥ 1 and ≤ −1). In *Δtplus3a-tplus3b/Df(2L)BSC151* testes, 398 transcripts were differentially expressed, of which 209 were down-regulated and 189 were up-regulated compared to the wild-type (Fig 5A). In *tbrd-1*^*1*^ mutant flies, 1,499 genes were differentially expressed, of which 1,196 were down-regulated and 303 were up-regulated compared to the wild-type.

The number of genes down-regulated in both *Δtplus3a-tplus3b/Df(2L)BSC151* and *tbrd-1*^*1*^ mutants, namely 141, represents about two-thirds of the genes that are activated by tPlus3a and tPlus3b (Fig 5A). On the other hand, only 12% of the differentially expressed genes in *tbrd-1*^*1*^ mutants overlap with genes expressed in *Δtplus3a-tplus3b/Df(2L)BSC151*, which suggests that most genes regulated by tPlus3a and tPlus3b are also targeted by tBRD-1, but that tBRD-1 itself activates a much larger set of genes. Of the up-regulated genes in *Δtplus3a-tplus3b/Df(2L)BSC151* and *tbrd-1*^*1*^ mutants, 56 overlap, which corresponds to about 30% of the tPlus3a and tPlus3b up-regulated genes and 17% of the genes up-regulated in *tbrd-1*^*1*^ mutants. Our results suggested that bromodomain proteins specifically synthesized in the testis serve as regulators of different sets of target genes in the germ line transcription machinery. Therefore, we also compared our RNA-seq data of *Δtplus3a-tplus3b/Df(2L)BSC151* and *tbrd-1*^*1*^ mutants to RNA microarray data from *tbrd-2* knock-down testes [12]. In contrast to the large number of target genes common to tPlus3a and tPlus3b and tBRD-1, the expression of only six genes changed in both the absence of tPlus3a and tPlus3b and upon knock-down of tBRD-2. Four of these genes are also regulated by tBRD-1; 66 genes are regulated by both tBRD-1 and tBRD-2 (Fig 5B). These findings suggest a role for tPlus3a and tPlus3b in defining a subset of genes regulated by tBRD-1.

To validate our RNA-seq results, we performed real-time quantitative PCR (qPCR) with cDNA from wild-type and *Δtplus3a-tplus3b/Df(2L)BSC151* and *tbrd-1*^*1*^ mutants. We first validated down-regulation of *tbrd-1* and *tplus3a-tplus3b* in the respective mutants and observed a clear decrease in the number of the transcripts expressed. *tplus3a-tplus3b* transcripts in *tbrd-1*^*1*^ mutants were also visibly down-regulated. According to our RNA-seq analysis, *tplus3a-tplus3b* transcripts were reduced by 40–45% in *tbrd-1*^*1*^ mutants; these transcripts do not appear in the results owing to a less than twofold decrease in the log2-fold change (Fig 5C). We determined the transcript levels of the down-regulated target genes *CG7542*, *CG2772*, and *kl-3*. Our results confirmed that *CG7542* is down-regulated in both mutants, and *CG2772* and *kl-3* are down-regulated only in *Δtplus3a-tplus3b/Df(2L)BSC151* testes (Fig 5C). We then validated the results for the up-regulated genes *Sox21b*, *Hsp67Bc*, *Hsp70Aa*, *CG13428*, and *AstCC* (Fig 5C). *Hsp67Bc* was slightly up-regulated only in *Δtplus3a-tplus3b/Df(2L)BSC151*, *Sox21b* was up-regulated, and *Hsp70Aa* was strongly up-regulated in both *Δtplus3a-tplus3b/Df(2L)BSC151* and *tbrd-1*^*1*^ mutants.

The top 40 most down- or up-regulated genes in both *Δtplus3a-tplus3b/Df(2L)BSC151* and *tbrd-1*^*1*^ mutant testes or in only *Δtplus3a-tplus3b/Df(2L)BSC151* testes are given in Table 1 (highest logFC as top).

**Table 1:** Genes down-regulated by tPlus3a and tPlus3b (from -7,1 to -1,3 log2FC) and by both tPlus3a and tPlus3b and tBRD-1 (from -8,1 to -2,7 log2FC). Genes up-regulated by tPlus3a and tPlus3b (from 6,2 to 1,7 log2FC) and by both tPlus3a and tPlus3b and tBRD-1 (from 4,0 to 1,3).

Down-regulated genes		Up-regulated genes	
*Δtplus3a-tplus3b/ Df(2L)BSC151* only	*Δtplus3a-tplus3b/ Df(2L)BSC151* and *tbrd-1*^1^	*Δtplus3a-tplus3b/ Df(2L)BSC151* only	*Δtplus3a-tplus3b/ Df(2L)BSC151* and *tbrd-1*^1^
CG31703	Salt	CG34436	Sfp87B
CG31702	CG14245	CG13428	Peb
Muc30E	Muc11A	AstCC	Sfp24Bb
CG2772	CG7542	CG12990	Sox21b
CG43401	Mur18B	Elo68alpha	PebII
Dh31-R	CG16762	CG10764	Obp56g
CG9259	CG8028	CG43320	Sfp60F
CG42370	CG3285	Uro	CG17572
CG6293	CG18095	CG18180	CG14304
CG12017	CG34426	CG12268	CG3868
CG12009	CG32023	Acp24A4	ppk19
CG43707	CG45072	CG34113	CG30427
dpr7	CG14292	Eip63F-1	Hsp70Aa
CG41434	CG31198	GstZ2	Hsp70Ab
IM23	CG3290	CG42313	AttB
kl-3	Irk3	CG11951	Hsp70Ba
CG34393	CG42235	kek3	Hsp70Bb
CG8641	Smvt	ninaC	Hsp70Bc
Ory	unc80	CG4267	Hsp70Bbb
CG8093	CG2187	CG14535	CG11893
mthl8	Scp2	rho-6	CG2065
IM1	CG34284	CG13038	Hsp67Bc
CG31897	CG11626	CG2663	CG18278
CG31600	CG13309	Snoo	CG1441
l(2)34Fc	CG2736	CG5697	CG30059
CG6967	CG14219	CG11598	CG10550
CG14982	Oatp58Da	CG31710	CG10096
CG8301	CG7882	asparagine-synthetase	CG10097
CG8145	NaPi-T	CG10657	fd102C
CG12869	CG10560	CG7214	CG5337
CG42368	CG34043	NimC2	CG31157
CG17162	CG9512	TwdlT	CG13285
CG16898	CG3690	CG16820	GstD5
CG11634	CG31202	SA-2	sisA
CG13748	CG31373	CG12780	Cyp4e3
stum	CG1809	CG45546	Arr1
CG34166	CG31106	Adh	Adgf-D
CG17159	CG5791	Adhr	CG6488
H15	SA	CG13516	CG31300
CG11629	CG7084	Rdl	CG31777

^1^: indicates the *tbrd-1* allele used for this studies

### tPlus3a and tPlus3b activate the Y-chromosomal fertility factors *kl-3* and *kl-5*

The set of genes down-regulated in *Δtplus3a-tplus3b/Df(2L)BSC151*, i.e., regulated by tPlus3a and tPlus3b, includes *kl-3* and *kl-5*, which encode Y-chromosomal fertility factors. These genes were not co-regulated by tBRD-1. As *kl-3* and *kl-5* encode dynein heavy chains of the axoneme, their down-regulation could contribute to the severely reduced fertility in *Δtplus3a-tplus3b/Df(2L)BSC151* flies that we observed.

### tPlus3a and tPlus3b and tBRD-1 negatively regulate heat shock genes and seminal fluid protein genes in the germ line

Strikingly, many heat-shock-related genes, e.g., *Hsp70Aa*, are among the genes up-regulated in both mutants (Table 1). Therefore, tPlus3a and tPlus3b and tBRD-1 might fulfill a repressive function for this gene class by limiting the activation of heat shock genes during the spermatocyte phase. Another class that appears to be regulated by both factors is represented by genes such as *Sfp87B* and *Sfp24Bb*, which encode seminal fluid proteins. These are synthesized by somatic parts of the reproductive tract and are first needed after sperm development [29]. *Acp24A4*, which encodes a male accessory gland protein [30], was only regulated by tPlus3a and tPlus3b. We found these transcripts up-regulated in all three biological samples (S5A Fig) and hardly any variation was found between the independent samples (S5A Fig). However, we asked if these transcripts could be up-regulated due to contaminations of testes preparations by accessory gland material. First, we searched for corresponding proteins in proteome data (containing 5500 proteins and recently generated in our group) from adult testes of wild-type males [24]. We did not detect accessory gland proteins in our proteome data which indicates clean testes preparation. As RNA-seq might be more sensitive we aimed at clarifying whether our testes RNA preparations were contaminated with transcripts from accessory glands. We searched for transcripts of other abundant seminal-fluid-relevant transcripts in our RNA-seq data. We searched for *Mst57Da*, *Mst57Db*, *Mst57Dc* [31], *Sfp96F* (FlyBase), and *GGT-1* [32]. We found that these genes were not regulated by tPlus3a and tPlus3b and/or tBRD-1 (S5B Fig). Transcripts for the ejaculatory bulb proteins Ebp and EbpII (FlyBase), however, were found to be repressed directly or indirectly by tPlus3a and tPlus3b and tBRD-1 (S5A Fig). The data for transcripts of somatic parts of the male reproductive system are summarized in Table 2.

**Table 2:** Up-regulation of transcripts in Δ*tplus3a-tplus3b* and *tbrd-1* mutant testes which are synthesized in somatic parts of the male reproductive track.

Transcript	Up-regulated in Δ*tplus3a-tplus3b* mutants	Up-regulated in *tbrd-1* mutants
*Acp24A4*	+	-
*Ggt-1*	-	-
*Mst57Da*	-	-
*Mst57Db*	-	-
*Mst57Dc*	-	-
*Ebp*	++	+
*EbpII*	++	+
*Sfp24Bb*	++	+
*Sfp60F*	++	+
*Sfb87B*	++	+
*Sfp96F*	+	-

- no up-regulation of transcripts, + clear up-regulation, ++ high level of up-regulation

These findings suggested that transcriptional repression of *Ebp*, *EbpII*, *Sfp24Bb*, *Sfp60F*, *and Sfp87B*, depended directly or indirectly on tPlus3a and tPlus3b and tBRD-1 in spermatocytes, while *Sfp96F* and *Acp24A* appeared solely regulated by tPlus3a and tPlus3b.

## Discussion

*D*. *melanogaster* spermatogenesis is characterized by a unique transcriptional program in spermatocytes. Thousands of transcripts are synthesized in this germ cell stage, but most are only needed much later in post-meiotic development [1–3]. Jiang et al. [33] reported that one-third of the tTAF-dependent genes is regulated by Modulo or Acj6. In our previous studies, we described a possible interplay between tBRDs and the testis transcription machinery and a role of tBRDs in guiding the complexes Mediator, tMAC, and TFIID to specific subsets of target genes [11–12]. We hypothesize that in addition to tBRD-1 and tBRD-2, other factors are required for fine-tuning gene expression during spermatogenesis. In the current study, we characterized synthesis and function of tPlus3a and tPlus3b. We show that tPlus3a and tPlus3b and the bromodomain protein tBRD-1 co-regulate distinct groups of target genes, which underscores their potential for fine-tuning the transcriptional regulatory network in spermatocytes. *tplus3a*-*tplus3b* are target genes of Aly and MED22 [8, 34], thus probably of the tMAC and Mediator complexes and are also slightly down-regulated in *tbrd-1*^*1*^ mutants, which indicates that regulators of transcription are interdependent.

### Plus3 domain proteins are synthesized in the testis and might be functionally redundant

The genome of *D*. *melanogaster* has the potential to synthesize only a few proteins containing a Plus3 domain. Here, we showed that the Plus3 domain proteins tPlus3a and tPlus3b are essential for full male fertility. In male germ cells, tPlus3a and tPlus3b proteins were limited to nuclei of spermatocytes and localize in the chromosomal regions. tPlus3a and tPlus3b proteins also localize to distinct spots in some regions of the nucleus, which might correspond to areas of Y-chromosomal lampbrush loops. The lampbrush loops harbor genes encoding male fertility factors, e.g., *kl-3* and *kl-5*. In contrast to the Plus3 domain protein Rtf1, tPlus3a and tPlus3b lack the histone modification domain, which suggests that tPlus3a and tPlus3b are not able to directly modify histones by themselves. The Plus3 domain might be needed for DNA binding, as described for human Rtf1 [23]. Therefore, tPlus3a and tPlus3b could be required as a platform to recruit other histone-modifying enzymes. Based on a C-terminal consensus sequence in Rft1, tPlus3a, and tPlus3b, we conclude that a Paf1C interaction domain like that in Rtf1 is also present in tPlus3a and tPlus3b, but interaction between tPlus3a and tPlus3b and members of Paf1C remains to be examined. Interestingly, all homologues of Paf1C subunits in *D*. *melanogaster* are also synthesized in the testis [5], which might point to an interaction of at least one of the Paf1C components with tPlus3a and/or tPlus3b. In addition, a role in spermatogenesis for the remaining Plus3 domain protein-coding gene, *CG12498*, should be considered. Based on structural homologies between tPlus3a, tPlus3b, and CG12498, it seems likely that these proteins would be able to functionally replace each other to a certain degree. Functional redundancy between tPlus3a and tPlus3b and CG12498 might be the reason for the increasing fertility of 8-day-old *Δtplus3a-tplus3b/Df(2L)BSC151* males and could also explain why younger *Δtplus3a-tplus3b/Df(2L)BSC151* males are not completely sterile. We consider the possibility that the highly similar Plus3 domain protein CG12498 can replace the loss of tPlus3a and tPlus3b by increasing the *CG12498* transcript level or translational activity. The truncation of the PI domain of CG12498 might lead to an inefficient replacement of tPlus3a and tPlus3b. This is in agreement with our observation that it takes days to rescue fertility defects, albeit not to the wild-type level. Furthermore, RTF1 with an intact PI domain might compensate.

### tPlus3a and tPlus3b proteins regulate Y-chromosomal genes essential for full male fertility

Generation of *Δtplus3a-tplus3b* deletion mutants resulted in cysts with abnormal individualization during sperm individualization. The inefficient individualization of sperm likely contributes to the reduced fertility of males. In our RNA-seq data of *Δtplus3a-tplus3b/Df(2L)BSC151*, the *kl-3* and *kl-5* genes were down-regulated. Both of these targets encode axonemal outer arm dynein heavy chains, which suggests a role in assembly of the axoneme and motility of sperm [35–37]. As flies bearing *kl-3* or *kl-5* deletions lose the outer dynein arm of the flagellar axoneme [38], down-regulation of *kl-3* and *kl-5* might explain our finding that *Δtplus3a-tplus3b/Df(2L)BSC151* mutants have a reduced fertility [39–40]. *kl-3* and *kl-5* are both transcribed in the spermatocyte Y-loops [41]. Three of these loop structures can be observed in spermatocytes during spermatocyte development [27]. According to our RNA-seq data, *kl-3* and *kl-5* are regulated by tPlus3a and tPlus3b but not by tBRD-1. This might point to a specific function of tPlus3a and tPlus3b in the regulation of these male fertility factors. Along these lines, co-localization of tBRD-1 and tPlus3a and tPlus3b was only observed in chromosomal regions, whereas speckles of tBRD-1 and distinct spots of tPlus3a and tPlus3b did not overlap.

### tPlus3a and tPlus3b and tBRD-1 co-repress heat shock genes in spermatocytes

Our RNA-seq data also suggest a repressor function of tPlus3a and tPlus3b, as many genes were up-regulated in *Δtplus3a-tplus3b/Df(2L)BSC151*. Strikingly, several genes seemed to be up-regulated also in *tbrd-1*^*1*^ mutant testes. One group of genes (*Sfp87B*, *Sfp24Bb*, and *Sfp60F*) encode several seminal fluid proteins (Sfp). These are needed for fertilization and can found in the ejaculate of male flies [42]. *Peb* (*Ebp*, *Peb-me*) and *PebII* (*EbpII*), which encode proteins of the ejaculatory bulb, are likewise not expressed in germ cells of wild-type flies. Their proteins are transferred together with sperm in the ejaculate of males to female flies during mating to prevent early remating [43–44]. These protein classes are first needed during fertilization; therefore, it is not surprising that a repression mechanism controls these genes in germ cells. As the general transcription rate is extremely high in the spermatocyte phase, tPlus3a and tPlus3b and tBRD-1 might prevent untimely transcription of sperm fluid genes.

Interestingly, *AstCC* and *CG13428* were upregulated in *Δtplus3a-tplus3b/Df(2L)BSC151* but strongly down-regulated in *tbrd-1*^*1*^ mutants. This might indicate that these genes are regulated oppositely by tPlus3a and tPlus3b and tBRD-1.

Another gene group repressed by both tPlus3a and tPlus3b and tBRD-1 are heat shock genes. Many *Hsp70* genes as well as *Hsp67Bc* were up-regulated in *Δtplus3a-tplus3b/Df(2L)BSC151* and *tbrd-1*^*1*^. This finding might explain why primary spermatocytes were reported to poorly respond to heat shock in spermatocytes [45].

### tPlus3a and tPlus3b share a large subset of target genes with tBRD-1 but not with tBRD-2

As previously reported, the transcription machinery in spermatocytes is supported by several interactions between various complexes, such as TFIID, tMAC, and Mediator [8]. Recent findings indicate how the specificity for different sets of genes might be achieved by cooperation of general transcription factors with bromodomain proteins [11–12]. Our RNA-seq results for *tplus3a-tplus3b*-deficient fly testes and *tbrd-1*^*1*^ mutant testes suggest a role for Plus3 domain proteins in transcription regulation. Comparative analyses of our *Δtplus3a-tplus3b/Df(2L)BSC151* and *tbrd-1*^*1*^ RNA-seq datasets with microarray data from *tbrd-2* knock-down testis revealed that many genes are co-regulated by tPlus3a and tPlus3b and tBRD-1, whereas only a few genes are commonly regulated by tPlus3a and tPlus3b and tBRD-2. However, a direct comparison of microarray data and RNA-seq data should be handled with caution. A potential cooperation between tPlus3b and tBRD-1 is supported by their heterodimerization in the yeast two-hybrid system. We hypothesize that tPlus3a and tPlus3b proteins either activate or repress sets of target genes. In RNA-seq experiments, we cannot distinguish between direct binding to regulated genes or indirect action by regulation of other activation or repression relevant components. During gene activation tPlus3a and tPlus3b probably also interact with some factors of the general or testis-specific transcription machinery. Indeed, tBRD-1 heterodimerizes with the tTAF Sa [11].

**Fig 6:**
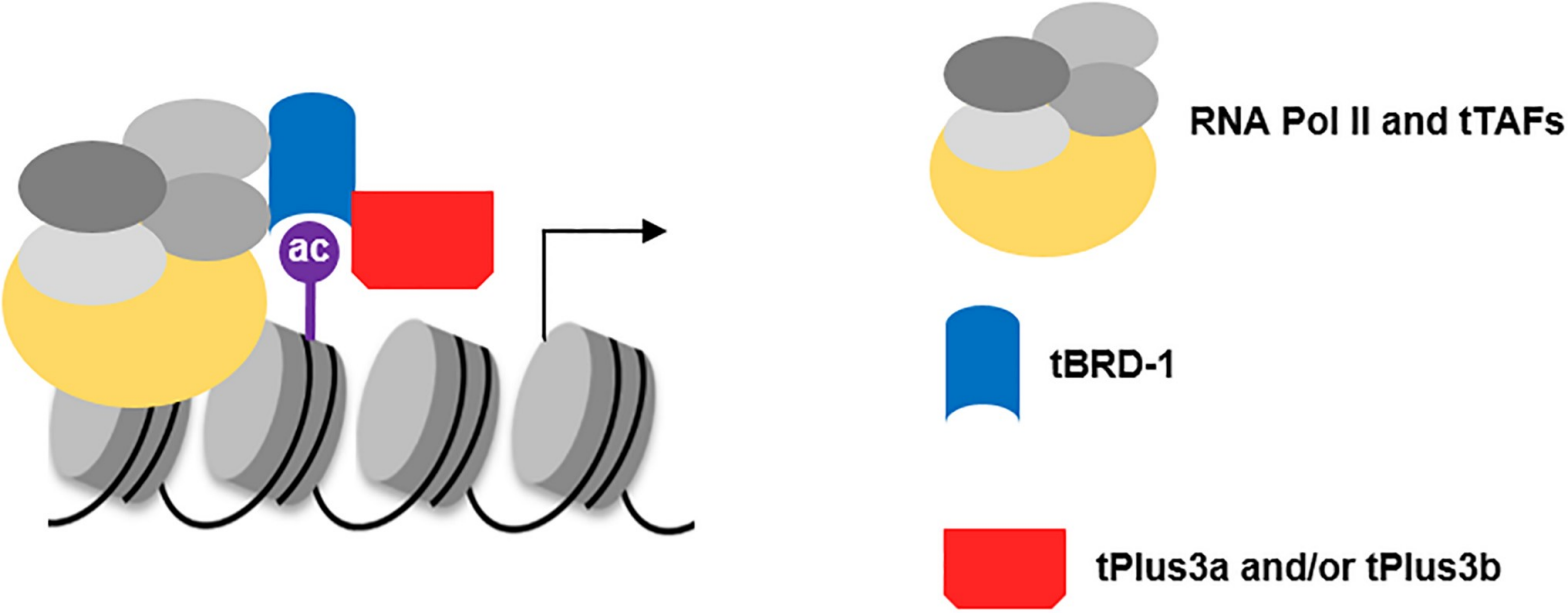
Model of the complex formation of tPlus3a and tPlus3b, tBRD-1, and other factors in gene activation. tPlus3a and tPlus3b molecularly interact with tBRD-1, which binds to acetylated histones. tBRD-1 interacts with the tTAF Sa to activate RNA Polymerase mediated transcription.

We previously showed that tBRD-1 binds to H3 peptides acetylated at lysines 9 and 14 and to H4 peptides acetylated at lysines 5, 8, and 12, which suggested a direct role of tBRD-1 in gene activation [12]. A small number of genes regulated by tPlus3a and tPlus3b and tBRD-1 and are also regulated by the tTAF Sa [46]. The genes regulated by all three proteins comprise ca. 12% of tPlus3a and tPlus3b-regulated genes, ca. 3% of tBRD-1-regulated genes, and ca. 1% of Sa-regulated genes. During transcription of these genes we propose a direct molecular interaction of Sa with tBRD-1, tBRD-1 with tPlus3a and tPlus3b, and tBRD1 binding to acetylated histones in an RNA Polymerase B containing complex (Fig 6).

## Material and methods

### Fly strains and cultures

Flies were maintained at 25 °C on standard medium. *w*^*1118*^ or *w*^*1118*^; *CyO/Sp; ProtB-eGFP* [47] flies were used as the wild-type control. *tbrd-1*^*1*^ mutants and flies synthesizing tBRD-1-eGFP [14] were used for RNA-seq and analysis of co-localization with tTPlus3a and tPlus3b, respectively. *w*^*1118*^; *Df(2L)BSC151/CyO* (Bloomington stock number: 9510) was used as deficiency fly line for *tplus3a* and *tplus3b*. *Δtplus3a and Δtplus3b* (*Δtplus3a-tplus3b*) mutants were maintained in a *y*^*1*^*cho*^*2*^*v*^*1*^ background, and *Δtplus3a-tplus3b/Df(2L)BSC151* trans-heterozygotes were made in a *w*^*1118*^ background. *w*^*1118*^; *Δtplus3a-tplus3b/Df(2L)BSC151; ProtB-eGFP* was used for visualization of nuclei phenotypic analyses.

### Generation of *tplus3b-eGFP* flies

The *tplus3b-eGFP* fusion construct was generated by PCR amplification of the *tplus3b* putative promoter region, 5’-UTR, and open reading frame (ORF) on *w*^*1118*^ genomic DNA with primers 5’-GGTACCTCGTGTAGCTCTTTTTAGC-3’ and 5’-ACTAGTGCCTTTCATTATCTCGTCCC-3’. The PCR product was subcloned into the pCRII-TOPO vector (Invitrogen). The recombinant plasmid was digested with *Kpn*I and *Spe*I and ligated into pChab*ΔsalΔlacZ* (modified after [48]), which carries the gene encoding eGFP, yielding a construct with *eGFP* at the C-terminus. Transgenic flies were established by injection into the *w*^*1118*^ strain.

### *In situ* hybridization

*In situ* hybridization of *w*^*1118*^ (wild-type), *y*^*1*^*cho*^*2*^*v*^*1*^; *Δtplus3a-tplus3b* and *y*^*1*^*cho*^*2*^*v*^*1*^; *Δtplus3a-tplus3b/CyO* was performed as described in Morris et al. [49]. *tplus3a-tplus3b* RNA probes were synthesized by PCR amplification of 610 bp of the *tplus3b* ORF with primers 5’-CAAATAAACTGGAGTCTGGTGG-3’ and 5’-TTGGCGATGTCATTAAGCGTTG-3’. The amplified product was cloned into the pCRII-TOPO vector (Invitrogen). Genes were transcribed *in vitro* for probe synthesis using SP6 and T7 polymerase (Roche) after linearization of the vector with *Kpn*I or *Eco*RI, respectively. Probes were then precipitated with ethanol and dissolved in hybridization solution [49]. *CG12498* and *rtf1 In situ* hybridization was performed using probes, which are based on constructs synthesized with the respective primers given in S1 Table.

### Immunofluorescence staining and antibody generation

Immunofluorescence stainings were performed according to Hime et al. [50] with modifications [51] DNA was stained with Hoechst 33258, and actin components, e.g., the individualization complex, were stained with TRITC-phalloidin. A tPlus3a and tPlus3b peptide antibody was raised against peptide KRTAKPEQHELEKYMRRKY (aa 329 to aa 347 of tPlus3a and tPlus3b) in rabbit, affinity purified (Pineda Antibody Service, http://www.pineda-abservice.de), and diluted 1:500. The secondary antibody anti-rabbit-Cy3 (Dianova) was diluted 1:100. Stainings were analyzed with an AxioPlan2 microscope (Zeiss). Images were processed using Photoshop (Adobe).

### RNA-interference and CRISPR/Cas9 mutant generation and analysis

For initial *tplus3a-tplus3b* functional studies the RNAi line 109981 (*P{KK115200}VIE-260B*, Vienna *Drosophila* Resource Center) was applied. For the *tplus3 and tplus3b* knock down, v109981 males were crossed against virgin females of *bam-Gal4/bam-Gal4*; *Sp/CyO*; *bam-Gal4-VP16/MKRS* (Bam). Crossings for knock down (Bam>>109981) experiments were maintained at 30 °C in parallel with 109981 and Bam controls.

*y*^*1*^*cho*^*2*^*v*^*1*^; *Δtplus3a-tplus3b* mutants were generated using a single target construct in the CRISPR/Cas9 system as described by Kondo and Ueda [28]. For cloning of the target construct, primers 5’-CTTCGCAGAATGGATGAGCGCTTA-3’ and 5’-AAACTAAGCGCTCATCCATTCTGC-3’ were used, and the product was ligated to *pBFv-U6*.*2*. The target construct was injected into *y*^*2*^*cho*^*2*^*v*^*1*^*P{nos-phiC31\int*.*NLS}X;attP2(III)*, and established transgenic flies were crossed with *y*^*2*^*cho*^*2*^*v*^*1*^;*attP40{nos-Cas9}/CyO* flies for mutagenesis. Fifty founder flies were crossed with *y*^*2*^*cho*^*2*^*v*^*1*^; *Sco/CyO* flies to establish potential mutants. Genomic regions next to the target sequence of homozygous offspring were analyzed by PCR and screened for deletions. Flies with *tplus3b-eGFP* in homozygous *Δtplus3a-tplus3b* situation were analyzed in fertility assays.

### Western blotting

Protein extracts for western blots were prepared from 20 testes of *w*^*1118*^, *y*^*1*^*cho*^*2*^*v*^*1*^; *Δtplus3a -tplus3b/CyO* and *y*^*1*^*cho*^*2*^*v*^*1*^; *Δtplus3a-tplus3b* flies. Testes were homogenized with 20 μl 2×SDS sample buffer by sonication for 20 min at 4 °C. Protein extracts were incubated for 5 min at 95 °C and separated on SDS-10% polyacrylamide gels. Western blotting followed standard procedures. Anti-tTPlus3a and tPlus3b was diluted 1:1,000 in 5% dry milk powder/1×TBS. HRP-conjugated anti-rabbit (Jackson Immunology) was diluted 1:1,000 in 5% dry milk powder/1×TBS. Enhanced chemiluminescence (ECL, Invitrogen) was detected on an Odyssey Fc Imaging System (Licor) following the manufacturer’s instructions.

### Sterility tests

For sterility tests, individual 1-day (after hatching)-old males (*w*^*1118*^ (wild-type), *w*^*1118*^;*Df(2L)BSC151/CyO*, *y*^*1*^*cho*^*2*^*v*^*1*^; *Δtplus3a-tplus3b/CyO* and *w*^*1118*^; *Δtplus3a-tplus3b/Df(2L)BSC151*) and 5-day (after hatching)-old *w*^*1118*^; *Δtplus3a-tplus3b/Df(2L)BSC151* males were placed with two virgin *w*^*1118*^ females in separate vials at 25 °C and were allowed to mate for three days. The final age of the flies was 4 days and 8 days, respectively. Sterility test for *tplus3a and tplus3b* knock down was performed at 25 °C using males which were grown at 30 °C (Bam, 109981 controls and knock down Bam>>109981). The number of offspring per vial was counted after 2 weeks and the mean is depicted with standard deviation (SD). Data were statistically analyzed using one-way ANOVA with Tukey’s multiple comparison test and Graph Pad Prism Version 5.

### Yeast two-hybrid experiments

Yeast two-hybrid experiments were carried out using the Matchmaker GAL4 Two-Hybrid System 3 (Takara Clontech) according to the manufacturer’s instructions. For construct generation, the *tplus3b* ORF of genomic *w*^*1118*^ DNA was PCR amplified with primers 5’-CCATGGCGATGGATGAGCGCTTAC-3’ and 5’-GTCGACCTAGCCTTTCATTATCTCGTC-3’; the products were cloned into the pCRII-TOPO vector (Invitrogen). The prey vector pGBKT7 was digested with *Nco*I and *Sal*I, and the bait vector pGADT7 was digested with *Nco*I and *Xho*I. The *tplus3b* ORF was excised from pCRII-TOPO with *Nco*I and *Sal*I and ligated into pGBKT7 for synthesis of the DBD-tPlus3b fusion protein (fusion with GAL4-DNA-binding domain) and into pGADT7 for synthesis of the AD-tPlus3b fusion protein (fusion with GAL4-activation domain). Yeast two-hybrid constructs for *tbrd-1* and *tbrd-2* are described in Theofel et al. [11].

### RNA isolation and RNA-seq

Total RNA from 200 testes of *w*^*1118*^; *tbrd-1*^*1*^ and *w*^*1118*^; *Δtplus3a-tplus3b/Df(2L)BSC151* flies was isolated using TRIzol (Invitrogen). DNA was digested with TURBO DNase (Invitrogen) and purified with the RNeasy Mini Kit (Qiagen) according to the manufacturer’s instructions. Transcriptomes were analyzed using next-generation sequencing (RNA-seq) in three replicates. Prior to library preparation, RNA quality was assessed using the Experion RNA StdSens Analysis Kit (BioRad). Libraries were prepared using the TruSeq Stranded mRNA LT Kit (Illumina) according to the manufacturer’s instructions. The quality of libraries was controlled using a Bioanalyzer 2100 and the Agilent High Sensitivity DNA Kit (Agilent). Pooled libraries were quantified with digital PCR (QuantStudio 3D, Thermo Fisher) and sequenced on the HiSeq 1500 platform (Illumina) in rapid-run mode with 50-base single reads. Reads were aligned to the *D*. *melanogaster* genome retrieved from Ensembl revision 89 (BDGP6) with STAR 2.4.1a [52]. Default parameters were used, except for outFilterScoreMin and outFilterMatchNmin, which were set to 60% of the read length. For analysis of RNA-seq data, tag counts were calculated and normalized to one million mapped exonic reads and gene length (FPKM). To generate the set of expressed genes, only genes with a minimum read count of 50 and a minimum FPKM of 0.3 were kept. Fold change was calculated between the technical replicates of two conditions. Differential expression was assessed using the log2 of the median fold change. Only genes with an increase or decrease of at least twofold were considered to be differentially expressed.

### PCR and qPCR

RNA from 200 testes of *w*^*1118*^; *tbrd-1*^*1*^ and *w*^*1118*^; *Δtplus3a-tplus3b/Df(2L)BSC151* was isolated using TRIzol (Invitrogen). DNA was digested with TURBO DNase (Invitrogen) and purified with RNeasy Mini Kit (Qiagen) according to the manufacturer’s instructions. RNA (1 μg) was used for reverse transcription with random hexamer primers using the Transcriptor First Strand cDNA Synthesis Kit (Roche). The qPCR reaction (20 μl) contained 10 μl iTaq Universal SYBR Green Supermix (Bio-Rad), 12.5 ng cDNA, and 10 μM gene-specific forward and reverse primers (S1 Table), and was run on an Agilent Stratagene Mx3000P cycler with an annealing temperature of 60 °C. Ct-values for three technical replicates were normalized to the expression level of Rpl32. Depicted is the mean value and standard error (SE) of three replicates using GraphPad PRISM version 5.03. Statistical analysis was calculated with Student’s t-test and Bonferroni correction.

PCR for amplification of flanking genes was performed using primers for the respective genes listed in S1 Table. DNA was isolated from homozygous *y*^*1*^*cho*^*2*^*v*^*1*^; *Δtplus3a-tplus3b* males via S-Fly PCR.

### ArrayExpress accession data

RNA-seq data were deposited in the ArrayExpress [53] database at EMBL-EBI (www.ebi.ac.uk/arrayexpress) under accession number E-MTAB-7013.

## Supporting information

S1 Fig High conservation of Plus3 domains and protein interaction (PI) domains of male germ line expressed Plus3 domain proteins. (A) Shown are the Plus3 domain of *Drosophila* Rtf1 (dRtf1), tPlus3a and tPlus3b and CG12498. The three conserved positively charged amino acids that gave the Plus3 domain its name (Plus3) are marked in green and with *, other conserved amino acids are marked in grey. (B) PI domains of human RTF (hRTF), see [21]), *Drosophila* Rtf1 (dRTF1), and tPlus3a and tPlus3b. The region corresponding to the putative protein interaction domain (PI) is truncated in CG12498. Conserved amino acids in all shown proteins are marked in yellow, those conserved between the *Drosophila* Plus3 domains are marked in grey, those conserved only between dRtf1 and CG12498 are marked in blue.
(TIFF)

S2 Fig Transcript distribution of *CG12498* and *rtf1* in male germ cells. (A) *CG12498* transcripts were present mainly in stages before meiotic divisions. (B) Sense control for *CG12498*. (C) *rtf1* transcripts were visualized in spermatocytes and in post-meiotic stages. (D) Sense control for *rtf1*.
(TIF)

S3 Fig tPlus3b-eGFP is mainly expressed in the nucleus and nucleolus of spermatocytes. Whole-mount preparations of testis from flies expressed tPlus3b-eGFP (A) in the nucleoplasm of spermatocytes (arrow) and in the nucleolus (arrowhead). (B) tPlus3b-eGFP is expressed in the nucleus of round spermatids. Asterisk marks the hub region Scale bar = 20 μm.
(TIF)

S4 Fig Sperm accumulate in seminal vesicles after 5–6 days. (A B) ProtB-eGFP marked sperm in seminal vesicles of wild-type males. (D, E) ProtB-eGFP marked sperm accumulate in seminal vesicles of *Δtplus3a-tplus3b/Df(2L)BSC151* males.
(TIF)

S5 Fig tPlus3a and tPlus3b- and/or tBRD-1-dependent repression of genes, which are expressed in the somatic parts of the reproductive track. Three biological samples were prepared and analyzed by RNA-seq. Only little variation within an individual genotype was observed. The data for genes, which are transcribed in somatic parts of the reproductive tract are analyzed for loss of repression in the male germ line (A). Transcripts levels increased in mutants; FPKM values between 0 and 50, the w1 data were set to 0. (B) Transcript levels do not increase in mutants; FPKM values between 100 and 4000.
(TIF)

S1 Table Primers for PCR Experiments relevant for qPCR, in situ hybridisation probes and S-Fly PCR. (PDF)
