# Supplementary figures and images for: Drosophila melanogaster tPlus3a and tPlus3b ensure full male fertility by regulating transcription of Y-chromosomal, seminal fluid, and heat shock genes
Source: PLoS One. 2019 Mar 7;14(3):e0213177. doi: 10.1371/journal.pone.0213177 (PMC6405060; doi:10.1371/journal.pone.0213177)

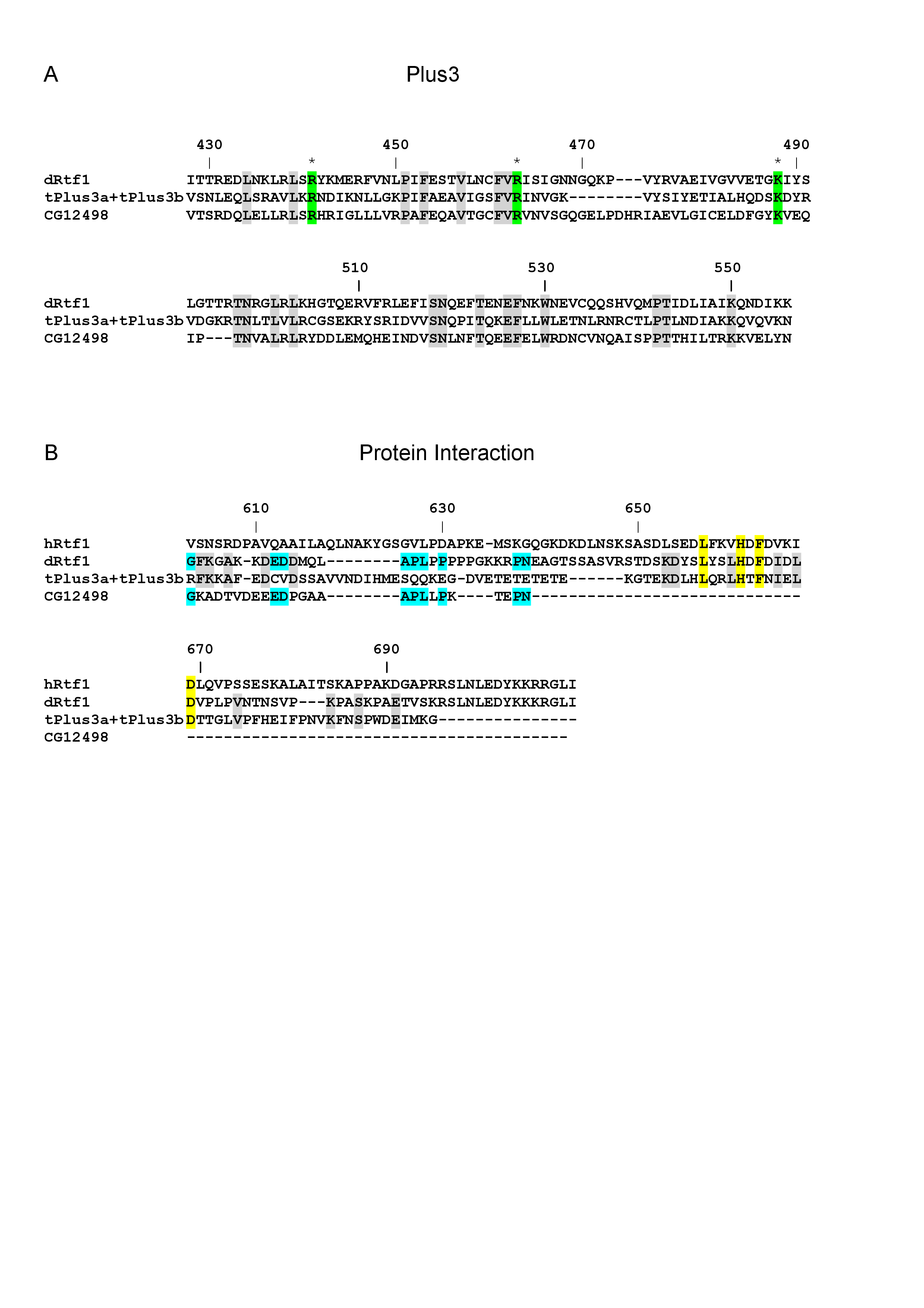

Supplement: S1 Fig — (A) Shown are the Plus3 domain of Drosophila Rtf1 (dRtf1), tPlus3a and tPlus3b and CG12498. The three conserved positively charged amino acids that gave the Plus3 domain its name (Plus3) are marked in green and with *, other conserved amino acids are marked in grey. (B) PI domains of human RTF (hRTF), see [21]), Drosophila Rtf1 (dRTF1), and tPlus3a and tPlus3b. The region corresponding to the putative protein interaction domain (PI) is truncated in CG12498. Conserved amino acids in all shown proteins are marked in yellow, those conserved between the Drosophila Plus3 domains are marked in grey, those conserved only between dRtf1 and CG12498 are marked in blue. (TIFF) [file pone.0213177.s001.tiff]
