# Supplementary material for: Drosophila melanogaster tPlus3a and tPlus3b ensure full male fertility by regulating transcription of Y-chromosomal, seminal fluid, and heat shock genes
Source: PLoS One. 2019 Mar 7;14(3):e0213177. doi: 10.1371/journal.pone.0213177 (PMC6405060; doi:10.1371/journal.pone.0213177)
Supplement: S1 Table — (PDF) [file pone.0213177.s006.pdf]

Suppl. Table 1: Primers

qPCR

Gene	Forward primer	Reverse primer
Rpl32	ATGACCATCCGCCCAGCATAC	CTGCATGAGCAGGACCTCCAG
tplus3a-tplus3b	AACATGAGTTGGAGAAGTATATGC	CTTCACATTCGGGAAAATTTCATG
tbrd-1	CGAAAGCGCAAGCAGGTTACATG	GGCAATTAACTCCCTGTACTTGGG
CG7542	TCCGAGGAGGGTCAAGAAAG	GAGGCATAATGGGCATCTCC
CG2772	TTGCTGCCCCAGAACGCG	GGCTCTGACACGCCGTAG
kl-3	AGATATGGGTCGGGCCTTAG	GGCATAGCCGGGATTCATAG
Sox21b	GCCACCACAGTGCTCCAG	GAAGTCTCGACCAGACCATG
Hsp67Bc	TCCATCTGGATGTGGGACTC	AAGAGATCCGATGGACCCAC
Hsp70Aa	ATGTGCTTATCTTCGACTTGGG	AAACAGTGCGTCAATCTCGATG
CG13428	AAGCTACTCTGCGTTGTTTTGG	TAGAGAAGGAACTGGGGTGG
AstCC	AACATTTTAAGTGGCAAATTCGGC	CTGACGGCATTGAAATAGCAAC

In-situ hybridisation probe templates

Gene	Forward primer	Reverse primer
tplus3a-tplus3b	CAAATAAACTGGAGTCTGGTGG	TTGGCGATGTCATTAAGCGTTG
CG12498	ATGTCTAACCAGCGATCCCG	TTCCTCCTCGTCTACGGTAT
rtf1	CTGATGTCGCTCGCCAAGAA	CTTGCCCTCTCTTTTGGCTT

S-Fly PCR of tplus3a-tplus3b flanking genes

Gene	Forward primer	Reverse primer
ttm3	CTGTCTTCTGAGTCCATGCG	CGACTGCACTGCTGAAGAAAG
not annotated gene	CGGAACCGACATATCTGCTG	AGTCCACCTCCTGTGCTTG
CG10834	TGGACCAGACGCTTTCTCAAC	GCGGAAATCGAGGATTTGCTG
CG42597	AAGATTTCTGTTTGGACGAAGGAC	AACGTCTATATACAGGCAATACCC
β3-tubulin control	ATCATTTCCGAGGAGCACGGC	GCCCAGCGAGTGCGTCAATTG
